# Biological wheat straw valorization: Multicriteria optimization of *Polyporus brumalis* pretreatment in packed bed bioreactor

**DOI:** 10.1002/mbo3.530

**Published:** 2017-10-27

**Authors:** Simeng Zhou, Isabelle Herpoël‐Gimbert, Sacha Grisel, Jean‐Claude Sigoillot, Michelle Sergent, Sana Raouche

**Affiliations:** ^1^ Aix‐Marseille Univ INRA BBF Biodiversité et Biotechnologie Fongiques Marseille France; ^2^ Aix‐Marseille Univ LISA Laboratoire d'Instrumentations et Sciences Analytiques Marseille France

**Keywords:** desirability function, D‐optimal design, fungal pretreatment, *Polyporus brumalis*, solid‐state fermentation, wheat straw

## Abstract

The purpose of this work was to optimize the pretreatment process of wheat straw by *Polyporus brumalis*_BRFM985 in order to improve carbohydrate accessibility for more efficient bioconversion. Indeed, there is growing demands to develop sustainable routes for lignocellulosic feedstocks valorization into value‐added products in energy, chemicals, materials, and animal feed fields. To be achieved, implementation of cheap and ecofriendly biomass pretreatment processes is necessary. In this frame, white rot basidiomycetes, well known for their ability to degrade lignin efficiently and selectively, are of great interest. The pretreatment of wheat straw by *Polyporus brumalis*_BRFM985 was performed in packed bed bioreactor and optimized using response surface methodology. The four pretreatment parameters optimized were metals addition (Cu, Mn, and Fe), time of culture, initial water content, and temperature. Multicriteria optimization highlighted that wheat straw pretreatment by *Polyporus brumalis_* BRFM985 in the presence of metals with high initial water content of 3.6 g H_2_O/g at 27°C for 15–16 days led to an improvement of carbohydrate accessibility with minimal matter loss.

## 1 INTRODUCTION

Lignocellulosic main polymers, that is, cellulose, lignin, and hemicelluloses, have tremendous potential as feedstocks for pulp and paper, biofuels, chemicals, nanoparticles, and animal feed productions (Beckham, Johnson, Karp, Salvachúa, & Vardon, 2016; Isroi et al., 2011; van Kuijk, Sonnenberg, Baars, Hendriks, & Cone, 2015). In many of these application fields, carbohydrate accessibility, which are naturally embedded in lignin matrix, is a main bottleneck. As a consequence, pretreatment is a crucial step in processes converting holocellulosic feedstocks to value‐added products. However, owing to the wide variety of lignocellulosic biomasses exhibiting diverse chemical compositions and structures, it is difficult to implement a unique pretreatment process (Alvira, Tomás‐Pejó, Ballesteros, & Negro, 2010). Various pretreatment processes were studied: biological, physical, chemical, and physicochemical ones (Alvira et al., 2010; Kumar, Barrett, Delwiche, & Stroeve, 2009). Among them, biological pretreatments, especially fungal pretreatment in solid‐state fermentation (SSF), have attracted interest due to simple techniques and equipments, low energy requirement, reduced downstream processing costs, and low pollution generation (Wan & Li, 2012).

Among the fungi, white rot (WR) fungi, from the phylum Basidiomycota, have been extensively studied mainly because they share the same primary objective with lignocellulosic valorization processes to break down lignocellulose into its monomeric constituents (Ohm et al., 2014). For this purpose, WR fungi produce a high number and broad variety of extracellular enzymes including hydrolytic enzymes as well as oxidative ones (Levasseur, Drula, Lombard, Coutinho, & Henrissat, 2013). These different, complementary, and synergistic catalytic activities utilized by WR fungi at the right time constitute an efficient lignocellulose degradation mechanism. Many WR fungi have been studied for biological pretreatment of various lignocellulosic biomasses (Liu et al., 2013; Mohanram, Rajan, Carrier, Nain, & Arora, 2015; Vasco‐Correa & Li, 2015) allowing holocellulose enrichment with a high delignification of the biomass. Within fungal species and strains, this ability depends of course on feedstock nature, but it also depends on the SSF culture conditions.

As a prerequisite for industrial implementation, biological pretreatment of lignocellulose with candidate strain, often selected at flask level, has to be performed in dedicated bioreactor and improved. This can be achieved through optimization of operating parameters such as time of culture, temperature, and water content (van Kuijk et al., 2015; Kumar et al., 2009; Tian, Fang, & Guo, 2012; Wan & Li, 2012). Carbohydrate accessibility increases with time of culture, but dry mass losses also. Then, the process efficiency has to be balanced with the matter losses. Water content of the substrate and temperature affect the fungal metabolism, notably the consumption of cell wall polymers (holocellulose, lignin) to different extent (Tian et al., 2012; Wan & Li, 2012). Moreover, the addition of inorganic salts has been reported as suitable way to improve the carbohydrate conversion yields (Salvachua, Prieto, Vaquero, Martinez, & Martinez, 2013; Song, Ma, Zeng, Zhang, & Yu, 2013; Wang, Yuan, Cui, & Dai, 2012). Owing to that, SSF pretreatment improvement through simultaneous optimization of relevant critical criteria, that is, dry mass losses, selectivity, and net carbohydrate accessibility, evaluated by net enzymatic hydrolysis yields, should allow the determination of the optimal operating windows for biological lignocellulose valorization.

In the present work, wheat straw fungal pretreatment by *Polyporus brumalis*_BRFM985 (Zhou et al., 2015) was carried out in bench‐top packed bed bioreactors under operating conditions approaching industrial practices. The purpose was to optimize the pretreatment process in order to improve carbohydrate accessibility, evaluated by net enzymatic hydrolysis yields, with minimal matter loss for a more efficient bioconversion. The four selected SSF parameters were metals addition (Cu, Mn, and Fe), time of culture, initial water content, and temperature (Kumar et al., 2009; Nakade et al., 2013; Salvachua et al., 2013; Sindhu, Binod, & Pandey, 2016; Song et al., 2013; Tian et al., 2012; Wang et al., 2012). Optimization was carried out using response surface methodology and multicriteria analysis with a D‐optimal design and desirability function approach.

## 2 MATERIAL AND METHODS

### 2.1 Biological materials sources

The strain *Polyporus brumalis*_BRFM985 was obtained from the “Centre International de Ressources Microbiennes,” fungal collection dedicated to filamentous fungi of biotechnological interest (CIRM‐CF; https://www6.inra.fr/cirm_eng/CIRM-CF) hosted by the National Institute of Agricultural Research (INRA), Marseille, France. The fungus was maintained on 2% (w/v) malt extract, 2% (w/v) agar (BD Difco) slants at 4°C. Naturally dried wheat straw (Haussmann soft wheat) was obtained from Vivescia (Reims, France) and chopped to ~4 mm (Cutting Mill SM 100, Retsch^®^, Germany).

### 2.2 Inoculum preparation and solid‐state fermentation

Fungal strains were cultivated for 7 days at 30°C on 2% (w/v) malt extract, 2% (w/v) agar (BD Difco, France) plates. Five 5 mm disks picked from the growth front of the plates were used to inoculate sterile Roux flasks containing 200 ml of medium (2% (w/v) malt extract) before incubating for 10 days at 30°C. Afterward, the mycelia mats were collected aseptically on Miracloth (Calbiochem, USA) and blended with sterile deionized water at 9500 rpm for 60 s using an Ultraturrax blender. The fungal suspension (12 ± 1 mg (dm) mycelia/ml) was used as inoculum for fungal pretreatment experiments.

Fungal pretreatment were carried out in packed bed bioreactor (250 ml glass columns; 20 cm × 4 cm, Legallais, France) system designed in a previous work (Meza, Lomascolo, Casalot, Sigoillot, & Auria, 2005). Briefly, 20 g dm of dry chopped wheat straw, wetted with 9 ml deionized water, were sterilized at 110°C for 30 min in autoclave bag and cooled at room temperature. Afterward, 10 ml of fungal inoculum suspension and when relevant metal stock solution (CuSO_4_, FeSO_4_, and MnSO_4_ 0.9 μmol each/g dm substrate) and sterile deionized water were directly added to the bag containing wheat straw. After homogenization by manual blending, the content of the bag was aseptically emptied in the sterile glass column and incubated in a controlled temperature water bath. For each bioreactor, the air stream was filter sterilized (0.2 μm) and wetted through a washing flask containing sterile deionized water before being distributed at a 0.5 v.v^−1^.m^−1^ flow rate. Regulation was done using a needle valve and flowmeter floating ball (R2‐15‐AA, Brooks). Noninoculated wheat straw was incubated under the same conditions and referred as control.

At the end of the culture, columns were emptied and their content was manually blended and aliquoted to be further analyzed.

### 2.3 Sample analysis

#### 2.3.1 Dry mass loss

One gram (wet basis) sample was dried at 105°C until constant mass to measure dry mass content and to estimate weight loss (final to initial dry mass percentage). The mean values (*n* = 3) are reported.

#### 2.3.2 Cell wall polymer composition analysis

Klason lignin and polysaccharide contents of control and biopretreated wheat straw were determined in duplicate according to NERL method (Sluiter et al., 2008). The untreated and the noninoculated wheat straw (control) used in this study consisted of almost 37% (w/w) cellulose, 27% (w/w) hemicelluloses, and 23% (w/w) lignin.

#### 2.3.3 Enzyme assays

Extracellular proteins were extracted from 2 g dm aliquots of biopretreated wheat straw with deionized water (5% w dm/v) for 1 hr at 4°C and 200 rpm. The extracts were recovered by filtration through GF/F filters (Whatman) and were stored at 4°C before analysis. Laccase and peroxidase activities were evaluated in the water extracts and expressed in international enzyme units per gram of dry matter sample (U/g dm) as described in a previous study (Zhou et al., 2015). All analyses were performed in triplicate.

#### 2.3.4 Enzymatic hydrolysis

Control or biopretreated wheat straw slurries with a consistency of 5% (w dm/v, in 50 ml Falcon^®^ tubes) were first subjected to a mild alkali treatment with 0.1% sodium hydroxide at 50°C and 120 rpm for 1 hr (Multitron, Infors AG, Switzerland). Afterward, the pH was adjusted to 4.8 by the addition of citrate phosphate buffer (pH 4, 100 mM) and supplemented with 12 FPU/g dm substrate of the commercial cellulase cocktail GC220 from *Trichoderma reesei* (Genencor Danisco, NY, USA) and 60 U/g dm substrate of β‐glucosidase SP188 from *Aspergillus niger* (Novozyme, Demark). Tetracycline (0.15 mg/ml) and cycloheximide (0.04 mg/ml) were added to prevent any microbial contamination. Then, the slurries consistencies dropped to 2.5% (w dm/v). The reaction was carried out at 50°C and 120 rpm for 72 hrs. The released reducing sugars and glucose were quantified at end point after centrifugation (5000 rpm, 5 min) using the dinitrosalicylic acid method and the Glucose RTU kit (Biomérieux, Marcy‐l’étoile, France), respectively.

The hydrolysis of the untreated and the noninoculated wheat straw (control) by the commercial enzymatic cocktail led to 25 and 23% (w/w) net cellulose and holocellulose conversion yields, respectively.

### 2.4 Experimental design and statistical analysis

The experimental design and the treatment of experimental results (multilinear regression, statistical tests, etc.) were performed with NemrodW^®^ software (Mathieu, Nony, & Phan‐Tan‐Luu, 2000). The models have been validated (lack of fit and regression test) by analysis of variance (ANOVA). The coefficient of determination (R^2^) and the adjusted one (adj‐R^2^) were used as a measurement of the proportion of total variation of outputs explained by the model. Afterward, graphical plots representative of the response surfaces in the experimental domain were drawn using NemrodW^®^ software.

Multicriteria optimization was achieved using the desirability function approach (Derringer & Suich, 1980). For each response *Y*_*i*_, a desirability function (*d*_*i*_), varying from 0%, outside of the satisfactory limit, to 100%, maximal satisfaction, is defined based on expert information. Afterward, an overall desirability function (*D*), based on the individual ones (*d*_*i*_), is constructed and then optimized. The overall desirability function *D* is defined as the weighted geometric average of *n* individual desirability functions as follows:

$$D=\sqrt[{n}]{{d_{1}}^{p_{1}}\times {d_{2}}^{p_{2}}\times \ldots \times {d_{n}}^{p_{n}}}$$

where *n* the number of responses to optimize, *p*_*i*_ (*i* = 1, 2, …, *n*) the weighting attributed to the *i*th response.

## 3 RESULTS AND DISCUSSION

The objective was to determine the optimal operating windows for the SSF pretreatment of wheat straw by *Polyporus brumalis*_BRFM985 allowing an improvement of cell wall carbohydrate accessibility. The four selected SSF operating parameters were metals addition (U_1_), time of culture (U_2_), initial water content (WM/DMi, U_3_), and temperature (U_4_). These parameters as well as their respective domain of variation (Table 1) were chosen on the basis of literature and preliminary experiments (Holker, Hofer, & Lenz, 2004; Pandey, 2003; Singhania, Patel, Soccol, & Pandey, 2009). To optimize the process, a modeling step was performed, taking into account the two disjoint domains (quantitative/qualitative) (Phan‐Tan‐Luu & Sergent, 2009). The global postulated model was:

$$Y_{i}=b_{0}+\sum_{i=1}^{n}b_{i}X_{i}+\sum_{\begin{array}{c} 1\leq i\leq n-1\\ i+1\leq j\leq n \end{array}}b_{ij}X_{i}X_{j}+\sum_{i=2}^{n}b_{ii}{X_{i}}^{2}$$

where *X*_*i*_ (*i* = 1, 2, …, *n*) the undimensional variables and b_i_ the coefficients of the model that will be estimated from the experimental results.

**Table 1:** Experimental domain and responses for the D‐optimal experimental design

	Factor	Undimensional variables	Level of factors	
			−1	+1
Qualitative variable	U_1_: Metals	*X*_1_	Yes	No
Quantitative variables	Factors		Experimental domain	
	U_2_: Time	*X*_2_	10 days → 20 days	
	U_3_: WM/DMi	*X*_3_	0.9 g H_2_O/g → 3.7 g H_2_O/g	
	U_4_: Temperature	*X*_4_	19°C → 31°C	

	Code	Full length name	Units
Responses	*Y*_1_	Mass loss	% (w/w)
	*Y*_2_	Cellulose loss	% (w/w)
	*Y*_3_	Holocellulose loss	% (w/w)
	*Y*_4_	Lignin loss	% (w/w)
	*Y*_5_	L/C loss	
	*Y*_6_	L/H loss	
	*Y*_7_	*Y*_cellulose^a^	% (w/w)
	*Y*_8_	*Y*_holocellulose^a^	% (w/w)
	*Y*_9_	Laccases	U/g dm
	*Y*_10_	MnP	U/g dm
	*Y*_11_	MiP	U/g dm

MiP: manganese‐independent peroxidase; MnP: manganese peroxidase.

a *Y*_: refers to net carbohydrate conversion yield.

To estimate the coefficients of this specific model, a D‐optimal design was selected (Figure S1), leading to a set of 18 experiments (Table 2). The center points of the quantitative variables experimental domain with level (−1) of the qualitative one were repeated four times (points 10 to 13) and were used to calculate the variance of experimental error and to test lack of fit of the postulated model. The objective of this modeling is to establish a quantitative relation between the variation in the outputs and the variation in the experimental conditions. Consequently, the coefficient will not be interpreted independently but through the fitting of the entire model with the results. Eleven responses regarding component losses (*Y*_1_ to *Y*_6_), net enzymatic hydrolysis carbohydrate yields (*Y*_7_ and *Y*_8_), and main ligninolytic activities (*Y*_9_ to *Y*_11_) were studied (Table 1). Relevant ones were selected to optimize wheat straw fungal pretreatment regarding targeted lignocellulose valorization fields.

**Table 2:** D‐optimal experimental design, experimental conditions, and responses

No. Exp	*X*_1_ (U_1_)	*X*_2_ (U_2_) (days)	*X*_3_ (U_3_) (g H_2_O/g)	*X*_4_ (U_4_) (°C)	*Y*_1_ (%)	*Y*_2_ (%)	*Y*_3_ (%)	*Y*_4_ (%)	*Y*_5_	*Y*_6_	*Y*_7_ (%)	*Y*_8_ (%)	*Y*_9_ (U/g dm)	*Y*_10_ (U/g dm)	*Y*_11_ (U/g dm)
1	−1 (YES)	0.5 (17.5)	0.87 (3.5)	0 (25)	19.3	26	24	46	1.7	1.9	32	30	0.5	1.67	0.17
2	−1 (YES)	−0.5 (12.5)	−0.87 (1.1)	0 (25)	13.3	27	21	25	0.9	1.1	13	13	4.4	0.05	0
3	−1 (YES)	0.5 (17.5)	−0.87 (1.1)	0 (25)	18.4	28	25	28	1.0	1.1	17	18	3.6	1.03	0.19
4	−1 (YES)	−0.5 (12.5)	0.87 (3.5)	0 (25)	15.8	25	23	36	1.4	1.6	24	23	0.3	1.28	0.13
5	−1 (YES)	0.5 (17.5)	0.29 (2.7)	0.82 (30)	25.4	33	36	53	1.6	1.5	33	31	0.6	1.31	0.17
6	−1 (YES)	−0.5 (12.5)	−0.29 (1.9)	−0.82 (20)	5.5	6	4	1	0.2	0.2	13	13	4.1	0.28	0.11
7	−1 (YES)	0.5 (17.5)	−0.29 (1.9)	−0.82 (20)	12.9	14	16	20	1.4	1.2	11	12	5.2	0.25	0.18
8	−1 (YES)	0 (15)	0.58 (3.1)	‐0.82 (20)	9.5	11	12	17	1.5	1.4	11	12	5.2	0.28	0.16
9	−1 (YES)	−0.5 (12.5)	0.29 (2.7)	0.82 (30)	18.6	25	23	39	1.6	1.7	28	25	0.9	1.45	0.14
***10***	−***1 (YES)***	***0 (15)***	***0 (2.3)***	***0 (25)***	***23.4***	***34***	***34***	***42***	***1.2***	***1.3***	***20***	***19***	***1.7***	***1.42***	***0.16***
***11***	−***1 (YES)***	***0 (15)***	***0 (2.3)***	***0 (25)***	***22.9***	***28***	***27***	***37***	***1.3***	***1.4***	***20***	***18***	***1.2***	***2***	***0.15***
***12***	−***1 (YES)***	***0 (15)***	***0 (2.3)***	***0 (25)***	***21.7***	***29***	***28***	***38***	***1.3***	***1.4***	***21***	***19***	***1.4***	***1.90***	***0.17***
***13***	−***1 (YES)***	***0 (15)***	***0 (2.3)***	***0 (25)***	***21.3***	***30***	***30***	***39***	***1.3***	***1.3***	***22***	***20***	***0.9***	***1.90***	***0.18***
14	1 (NO)	1 (20)	0 (2.3)	0 (25)	24.6	36	37	38	1.0	1.0	15	13	2.8	0.38	0.08
15	1 (NO)	−1 (10)	0 (2.3)	0 (25)	13.1	22	21	18	0.8	0.8	13	12	2.4	0	0
16	1 (NO)	0.5 (17.5)	0.87 (3.5)	0 (25)	20.4	25	28	38	1.5	1.4	22	19	1.3	0.61	0
17	1 (NO)	−0.5 (12.5)	−0.87 (1.1)	0 (25)	11.5	19	18	12	0.6	0.7	7	8	4.6	0.05	0
18	1 (NO)	0.5 (17.5)	−0.29 (1.9)	−0.82 (20)	8.6	21	20	17	0.8	0.9	11	11	5.1	0.23	0.10
19	1 (NO)	0 (15)	0.58 (3.1)	−0.82 (20)	10.7	14	9	13	0.9	1.4	9	10	7.0	0.14	0.15
20	1 (NO)	−0.5 (12.5)	0.29 (2.7)	0.82 (30)	20.3	24	23	31	1.3	1.4	18	16	3.5	0.13	0
21	1 (NO)	0 (15)	−0.58 (1.5)	0.82 (30)	20.8	27	28	32	1.2	1.2	9	8	3.4	0.29	0.13

*Y*_*i*_: refer to Table 1.

% are w/w ratio.

Bold and italic lines are repeated experiments at the center points of the quantitative variable experimental domain with level (−1) of the qualitative one.

### 3.1 Impact of SSF parameters on fungal growth and wheat straw chemical modifications

Fungal pretreatment of wheat straw by *Polyporus brumalis*_BRFM985 was performed in the controlled 250 ml packed bed SSF bioreactors using experimental conditions defined in Table 2. Fungal growth was evaluated by visual examination. White mycelial clusters could be observed early, on the second day of incubation, for almost all the tested conditions. While growth was delayed to day 3 to 5 for experiments performed at 20°C and/or with initial water content of 1.1 g H_2_O/g dm. Globally, it took almost 7 days to the fungus to fully colonize the wheat straw. Afterward, it grew extensively with thick and dense mycelial biomass. This led the substrate to be embedded in the mycelium network. For SSF performed at 20°C and/or with initial water content of 1.1 g H_2_O/g dm, wheat straw was fully colonized in almost 10 days but the mycelial biomass was tenuous and did not embed the wheat straw in its network. These observations are in accordance with literature as the lowest the temperature or water content, the slowest the fungal growth (Holker et al., 2004; Singhania et al., 2009). It is worth to note that an initial water content of 1.1 g H_2_O/g dm (the lowest one experimentally tested) already corresponds to a water activity of 1. Literature on SSF pointed out the fact that fungi have the advantage over bacteria to grow on “dry” substrate (Holker et al., 2004; Pandey, 2003). This is almost true for food spoilage fungi with water activity as low as 0.6 (Labuza, 1975). However, in the case of basidiomycetes, of which physiology differs drastically from ascomycetes, more available water seems to be needed and it fits with natural biotope.

Wheat straw degradation by the fungal pretreatment was evaluated by measurement of dry mass loss (*Y*_1_) and cell wall component (cellulose, holocellulose, and lignin) losses (*Y*_2_, *Y*_3_, and *Y*_4_, respectively). As expected from previous experiments, these four responses were highly correlated with coefficients of correlation ranging from 0.84 to 0.96.

**Figure 1:**
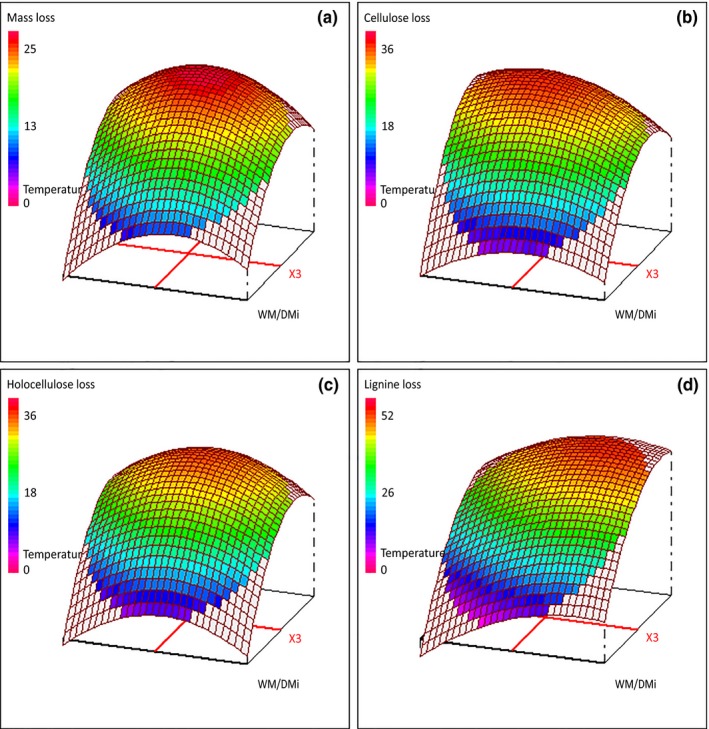
Response surface plots of variation of mass and component losses as a function of initial water content (*X*_3_) and temperature (*X*_4_). Metals addition (*X*_1_) and time of culture (*X*_2_) were, respectively, set to −1 (YES) and 0 (15 days). (a) Mass loss; (b) cellulose loss; (c) holocellulose loss; (d) lignin loss

Dry mass losses varied from 5.5 to 24.4% (w/w) in fungal pretreated samples (Table 2) against 2.6% (w/w) for the control one. The wide range of values can be explained by the experimental conditions allowing the fungus to have slow or fast growth accompanied by low or high metabolic activities. Salvachua and coworkers also obtained a wide range of weight losses values from 20 to 38% (w/w) when culturing *Irpex lacteus* on wheat straw with different SSF conditions (Salvachua et al., 2013). The data were well fitted by the model as shown by its high significance (*p* < .001) along with a R^2^ of 98.4% and 95.5% (adj‐R^2^) of total variation attributed to the independent factors (Table 3). Analysis of *Y*_1_ (Table 3 and Figure 1a) showed that time of culture (*X*_2_), initial water content (*X*_3_), and temperature (*X*_4_) had a significant impact and more particularly the temperature. Increase of mass loss with time of culture is obviously normal. As the fungus grows, it consumes available sources of nutrients on wheat straw leading to a decrease of dry mass. Same trends were observed when strains from the *Phlebia* genus were cultured on wheat straw for 10–30 days leading to approximately 2–17% (w/w) weight losses, respectively (Arora, Sharma, & Chandra, 2011). For the same time intervals, *Trametes versicolor* and *Pleurotus ostreatus* led to 6–24% and 15–31% (w/w) dry matter losses, respectively (Shrivastava et al., 2011). Regarding initial water content and temperature, their impact on mass loss were in accordance with literature (Singhania et al., 2009; Tian et al., 2012; Yoon, Ang, Ngoh, & Chua, 2014). It can be observed, on Figure 1a, an important increase of mass loss for temperature varying from 19 to 25°C, followed by a level off for temperature from 25 to 31°C. This suggested that catabolism optimum is at nearly 27–28°C with an initial water content of 2.3 g H_2_O/g dm. It is also well known that low or high moisture content has a detrimental effect on fungal physiology and metabolism in SSF culture and then induce lower mass losses (Tian et al., 2012). Water content too low means not enough solvent for reactions, while water content too high means high inertia in gas transfer (less oxygen readily available) and water soluble molecules dilution.

**Table 3:** Coefficients estimated by multilinear regression for the 11 responses

Terms	*Y*_1_	*Y*_2_	*Y*_3_	*Y*_4_	*Y*_5_	*Y*_6_	*Y*_7_	*Y*_8_	*Y*_9_	*Y*_10_	*Y*_11_
R^2^	0.984	0.937	0.929	0.972	0.944	0.976	0.979	0.989	0.927	0.940	0.929
Adj‐R^2^	0.955	0.820	0.797	0.921	0.841	0.932	0.941	0.969	0.793	0.829	0.799
B0	22.07	29.85	29.95	35.3	1.08	1.18	17.68	15.88	1.665	1.550	0.126
b1	−0.26	−0.40	0.20	−3.7^a^	−0.20^b^	−0.17^c^	−3.08^b^	−3.13^c^	0.365^a^	−0.255^a^	−0.039^b^
b2	5.46^c^	5.88^b^	8.38^b^	11.4^c^	0.28^b^	0.18^b^	2.75^a^	2.56^b^	−0.231	0.272	0.061^b^
b21	−0.24	1.38	0.88	−0.1	−0.12^a^	−0.10	−1.00	−1.69^a^	−0.281	−0.028	−0.021^a^
b3	1.77^a^	−1.07	−0.12	7.4^b^	0.36^c^	0.37^c^	7.04^c^	5.81^c^	−1.391^c^	0.304	−0.001
b31	0.96	0.06	−0.90	−0.6	−0.01	0.02	−0.26	−0.40	0.604^a^	−0.184	−0.022^a^
b4	8.25^c^	9.47^c^	10.27^c^	17.0^c^	0.35^c^	0.31^c^	6.42^c^	4.80^c^	−1.830^b^	0.338^a^	−0.009
b41	0.51	−2.53	−0.88	−2.2	0.01	−0.13^a^	−2.54^a^	−2.65^b^	−0.058	−0.227	−0.020^a^
b22	−2.96	−0.45	−1.15	−3.6	0.02	−0.11	−0.60	−0.25	0.570	−1.104^a^	−0.048^a^
b33	−6.46^c^	−6.08	−8.25^a^	−6.8	0.01	0.10	0.58	2.17	0.933^a^	−0.702^a^	−0.043^a^
b44	−7.75^c^	−12.62^b^	−12.65^b^	−12.5^b^	0.01	−0.06	−2.18	−0.92	2.812^b^	−1.155^b^	0.016
b23	−0.83	−2.14	−1.67	2.3	0.20^a^	0.11	1.24	0.14	0.445	−0.352	−0.091^b^
b24	0.40	−1.57	0.23	−5.0	−0.77^b^	−0.80^c^	3.14	3.47	−1.333^a^	0.154	0.028
b34	−2.03	−3.27	−0.81	−2.0	−0.42^b^	−0.57^b^	9.95^b^	8.72^b^	−2.396^a^	0.348	−0.077^a^

*Y*_*i*_: refer to Table 1.

a Statistically significant at the level 95% (*p* < .05).

b Statistically significant at the level 99% (*p* < .01).

c Statistically significant at the level 99.9% (*p* < .001).

Cellulose and holocellulose losses (*Y*_2_ and *Y*_3_) ranged from 6 to 36% (w/w) and 4 to 37% (w/w), respectively (Table 2). Correlation between *Y*_2_ and *Y*_3_ of 0.96 accompanied by a slope of 1 showed that both cellulose and hemicelluloses were consumed equally despite their differences in sugar residue composition. Balanced losses of both polysaccharides were also observed by Tuyen and coworker when culturing *Phanerochaete chrysosporium*,* Schizophyllum commune*, and *T. versicolor* on wheat straw for 49 days (Tuyen, Cone, Baars, Sonnenberg, & Hendriks, 2012). On the contrary, wheat straw pretreatment by *I. lacteus* led to unbalanced losses regardless of culture conditions or time (Salvachua et al., 2013). Such discrepancy could arise not only from the strain and the culture conditions but also from varietal or seasoning lignocellulose structure differences (Sindhu et al., 2016). As for *Y*_1_, the model fitted well the experimental data (Table 3) and the behavior of factors were the same: the most influential factor was temperature (*X*_4_), with a high increase for temperature varying from 20 to 25°C and a maximum at around 27°C (Figure 1b,c). Comments on the impact of the factors on both responses would be the same as for *Y*_1_ as fungal growth and catabolism might lead to the consumption of cellulose and hemicelluloses as primary source of carbon and energy.

Regarding lignin losses (*Y*_4_), values ranged from 1% to 53% (Table 2) and were in the range of reported date for 20 days or less wheat straw fungal pretreatment (Arora et al., 2011; Cianchetta, Di Maggio, Burzi, & Galletti, 2014; Salvachua et al., 2013; Shrivastava et al., 2011). Higher values were obtained by Tuyen and coworker but after 49 days of culture (Tuyen et al., 2012). The experimental data were well fitted by the model and the interpretation of the model showed an influence of the four factors (Table 3). It is well known from decades that WR fungi are able to degrade lignin into low‐molecular‐weight aromatic compounds partly metabolized by the fungus (Bugg, Ahmad, Hardiman, & Rahmanpour, 2011; Cragg et al., 2015; Eggert, Temp, & Eriksson, 1997). Delignification is a combination of enzymatic and chemical reactions especially involving metalloenzymes (laccases and peroxidases) and Fenton reactions (Tian et al., 2012). As expected from literature (Salvachua et al., 2013; Wang et al., 2012; Zeng, Singh, & Chen, 2011), addition of metals (Cu, Mn, and Fe) involved in the enzyme active sites or in the chemical reactions had a positive effect on delignification. Main ligninolytic activities (laccases, *Y*_9_, Mn peroxidases, *Y*_10_, and independent Mn peroxidases, *Y*_11_) were analyzed to bring insights into delignification process. Surprisingly, we observed that only peroxidases activities were positively impacted by metal addition (*X*_1_) (Table 3). The negative impact of metals on laccases cannot be explained, especially as a recent study has demonstrated that laccase expression is regulated by copper at the transcriptional level (Piscitelli et al., 2011). Regarding temperature and initial water content, response surface showed an influence of both parameters: lignin loss increased for high values of these factors (Figure 1d). More precisely, for temperatures from 20 to 25°C, lignin loss increased rapidly regardless of initial water content, but for temperatures from 25 to 30°C, it increased almost only with initial water content increase (Figure 1d). This is in accordance with thermodynamics of chemical and enzyme‐catalyzed reactions.

**Figure 2:**
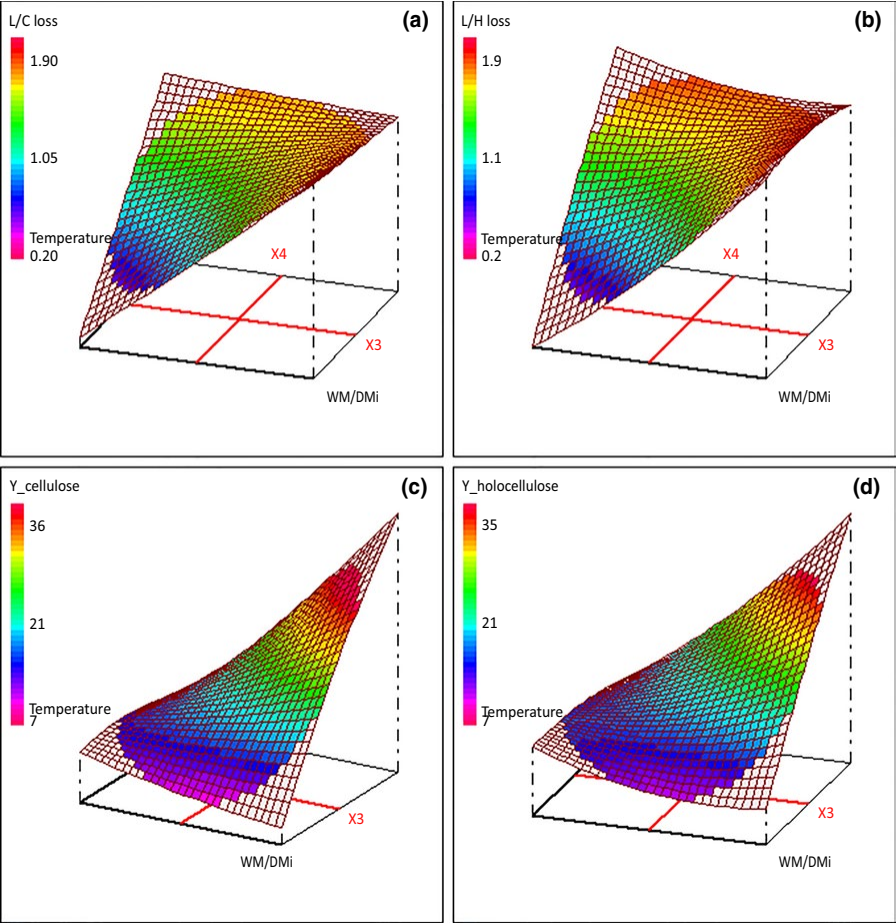
Response surface plots of variation of selectivity and of net enzymatic hydrolysis carbohydrate conversion yields as a function of initial water content (*X*_3_) and temperature (*X*_4_). Metals addition (*X*_1_) and time of culture (*X*_2_) were, respectively, set to −1 (YES) and 0 (15 days). (a) Lignin to cellulose loss selectivity; (b) lignin to holocellulose loss selectivity; (c) net cellulose conversion yield; (d) net holocellulose conversion yield

Selectivity is a good tool to evaluate the selective delignifying properties of a fungus. Lignin to cellulose losses ratio (L/C) and lignin to holocellulose losses ratio (L/H) selectivity (*Y*_5_ and *Y*_6_) values ranged from 0.2 to 1.7 and 0.2 to 1.9, respectively (Table 2). It is worth to note that the experimental conditions leading to selectivity values under 1.1 were those for which metals were not added, the temperature or the initial water content were set to 20°C or 1.1 g H_2_O/g dm. Experimental data were well fitted by the model despite a significant lack of fit at the level of 95% for *Y*_5_. All the four factors and almost all their interactions had a significant impact on both responses which are well correlated (0.95). Selectivity increased in the presence of metals (*X*_1_), which favor delignification, or with time of culture (*X*_2_), or initial water content (*X*_3_) or temperature (*X*_4_) (Table 3). However, due to factors interactions, carrying out SSF in the presence of metals at the highest temperature, initial water content or time of culture led to a decrease of both selectivity responses (Figure 2a, b). It could be explained by differential time course of lignin and holocellulose degradation during SSF culture as shown by Shi, Sharma‐Shivappa, Chinn, and Howell (2009).

### 3.2 Impact of SSF parameters on carbohydrate accessibility

Net cellulose and holocellulose conversion yields (*Y*_7_ and *Y*_8_) were evaluated as an indicator of carbohydrate accessibility. Their respective values ranged from 7 to 33% (w/w) and 8 to 31% (w/w) in fungal pretreated samples (Table 2) against 25 and 23% (w/w) for the control one. These data were in the range of reported ones, while the difference with the control is lower (Cianchetta et al., 2014; Salvachua et al., 2013). This could be explained by the enzymatic cocktail composition. The absence of additional xylanase activities in the enzymatic cocktail used in this work, as compared to Salvachua and Cianchetta group ones, might have led to an underestimation of both net cellulose and holocellulose conversion yields. Nevertheless, the model fitted well to the experimental data (Table 3) with all the four factors and almost all their interactions having a significant impact on both responses which are highly correlated (0.99) with a slope of almost 1. Net carbohydrate conversion yields increase in the presence of metals, with time of culture, initial water content, and temperature in the considered experimental domain (Figure 2c, d). Glycosyl hydrolases efficiency to release sugars from lignocellulosic biomasses totally relies on their accessibility to cellulose or hemicelluloses. This accessibility is hampered by the lignocellulose chemical and conformational structures (Alvira et al., 2010; Brodeur et al., 2011; Kumar et al., 2009). The fungal pretreatment, by its physical and chemical actions, should alter the lignocellulose structure improving enzymes accessibility and then sugars recovery from the remaining carbohydrates. This is highlighted by the good correlation between selectivity and net carbohydrate conversion yields of 0.68 and 0.70 between *Y*_7_ and *Y*_5_, and *Y*_8_ and *Y*_6_, respectively. The efficiency of the fungal pretreatment to increase carbohydrate accessibility has obviously to be balanced by mass losses to (*i*) be properly estimated and (ii) allow comparison with the control sample. To be strictly above control sample carbohydrate conversion yields, in the studied experimental domain, metals have to be added to wheat straw, time of culture, initial water content, and temperature have to be set to values higher than 12.5 days, 2.3 g H_2_O/g, and 25°C, respectively.

### 3.3 Multicriteria optimization

To optimize the SSF pretreatment of wheat straw by *Polyporus brumalis*_BRFM985 for improving cell wall carbohydrate accessibility and recovery, several responses have to be simultaneously optimized. The objective is to minimize mandatory dry mass losses while selectivity and net carbohydrate conversion yields are maximized. Unfortunately, it is unlikely that the optimum conditions reached for each factor would be the same for all responses. As a consequence, a compromise zone where all the experimental responses are satisfactory, have to be determined. This is achieved by multicriteria optimization using the desirability function approach (Derringer & Suich, 1980).

**Figure 3:**
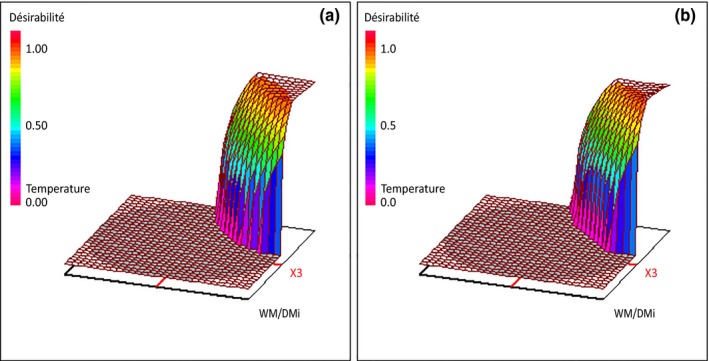
Response surface plots of variation of desirability functions as a function of initial water content (*X*_3_) and temperature (*X*_4_). Metals addition (*X*_1_) and time of culture (*X*_2_) were, respectively, set to −1 (YES) and 0 (15 days). (a) *D*_1_ desirability function; (b) *D*_2_ desirability function

In this work, two overall desirability functions were constructed either to optimize glucose recovery from cellulose (*D*_1_), or reducing sugars from holocellulose (*D*_2_) depending on the lignocellulose valorization field needs. The linear desirability functions were chosen to fulfill the objectives (Table S1). Dry mass losses (*Y*_1_) boundaries were selected based on literature and experimental data (Cianchetta et al., 2014; Salvachua et al., 2011) with an intermediate value of 15% (w/w) set as optimal (desirability *d*_1_ = 100%), while a loss above 25% (w/w) was not acceptable (desirability *d*_1_ = 0%). In the case of selectivity (*Y*_5_ or *Y*_6_), values should be higher than 1.1 to consider the fungal pretreatment as delignifying. Regarding net carbohydrate conversion yields (*Y*_7_ or *Y*_8_), values should be at least 10% higher than control ones to be considered as interesting. For the first overall desirability function (*D*_1_), *Y*_1_, *Y*_5_ and *Y*_7_ were took into account, while for the second one (*D*_2_), it was *Y*_1_, *Y*_6_, and *Y*_8_. As all the response variables were considered to have the same importance, the weights for the responses were set to 1. Results of both multicriteria optimizations are shown on Figure 3 and Table S1. As expected from the overall statistical analysis of the D‐optimal experimental design, optimal conditions were quite the same with the following setting: time of culture at almost 15 days, initial water content at 3.6 g H_2_O/g, and temperature at 27°C in the presence of metals. These settings were experimentally tested in triplicate. Dry mass losses were of 19.6% (w/w), while the predicted ones were around 19% (w/w). These values are in the range of reported data for beneficial pretreatments (Cianchetta et al., 2014; Salvachua et al., 2011). Lignin to cellulose and to holocellulose losses ratios were slightly higher than the predicted ones with 1.8 against 1.7, and 1.9 against 1.8, respectively. Nevertheless, these values are representatives of a high delignifying power. Regarding net carbohydrate conversion yields, 33 and 31% (w/w) were, respectively, obtained for cellulose and holocellulose against 34 and 32% (w/w) for the predicted ones. The experimental yields correspond to an improvement of cellulose and holocellulose conversion of 30% and 34%, respectively, as compared to control.

## 4 CONCLUSION

*Polyporus brumalis*_BRFM985 was used to pretreat wheat straw on bench top laboratory scale for lignocellulose valorization purposes. Four SSF culture parameters, metals addition, time of culture, initial water content, and temperature were studied to optimize this fungal pretreatment using response surface methodology involving a D‐optimal design. The experimental design was useful as it highlighted the nonlinear effect and the interaction effects between the four parameters, and the curvature in the domain. It also allowed to jointly improve carbohydrate accessibility with minimal matter loss. Metals (Cu, Mn, and Fe) have to be added in combination with an initial water content higher than 3.6 g H_2_O/g and the SSF culture has to be performed at 27°C for 15–16 days to achieve a more efficient bioconversion. As the next step to build‐up a successful biotechnological process, wheat straw pretreatment by *Polyporus brumalis*_BRFM985 should be performed at pilot/industrial scale within the defined optimal operating windows, and applied in bioenergy and animal feed fields. This should allow to take into account the impact of metals on the performance of the microbial consortia (biomethane) and on the nutritional aspect in animal diet.

## CONFLICT OF INTEREST

The authors declare no conflict of interest.
