# Supplementary figures and images for: Biological wheat straw valorization: Multicriteria optimization of Polyporus brumalis pretreatment in packed bed bioreactor
Source: Microbiologyopen. 2017 Oct 27;7(1):e00530. doi: 10.1002/mbo3.530 (PMC5822346; doi:10.1002/mbo3.530)

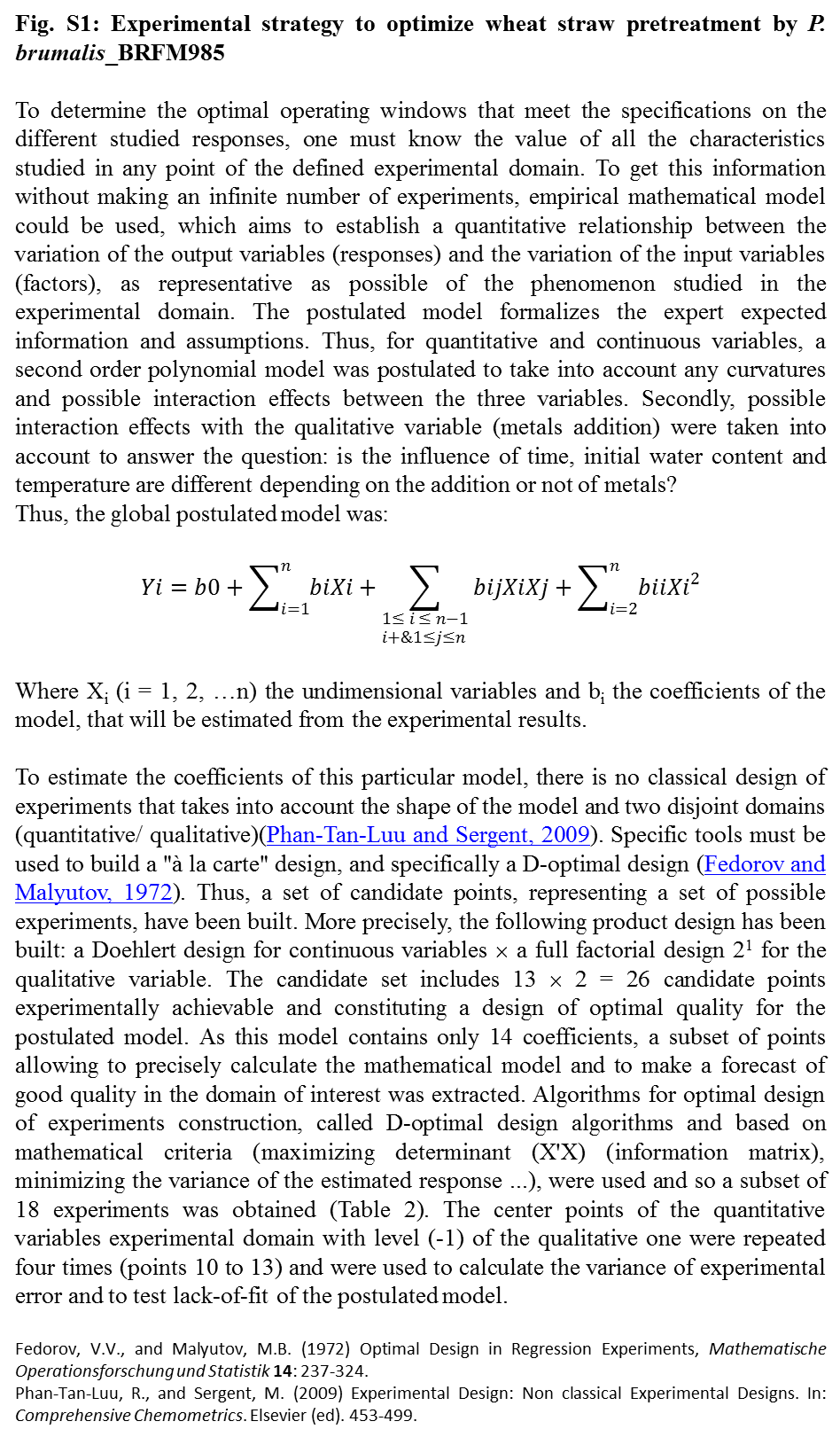

Supplement: Supplementary file 1 [file MBO3-7-na-s001.tif]
