# Supplementary material for: Biological wheat straw valorization: Multicriteria optimization of Polyporus brumalis pretreatment in packed bed bioreactor
Source: Microbiologyopen. 2017 Oct 27;7(1):e00530. doi: 10.1002/mbo3.530 (PMC5822346; doi:10.1002/mbo3.530)
Supplement: Supplementary file 2 [file MBO3-7-na-s002.pdf]

Table S1: Results of multi-criteria optimizations through desirability functions approach

		$D_1 = f(d_{Y1}; d_{Y5;} d_{Y7})$[a]			$D_2 = f(d_{Y1}; d_{Y6;} d_{Y8})$[b]		
Factors		Optimal Values					
U1	Metals	YES			YES		
U2	Time	15.7			15		
U3	WM/DMi	3.6			3.6		
U4	Temperature	27			27		
Responses		Predicted values	CI 95%[c]	d_i[d] (%)	Predicted values	CI 95%[c]	d_i[d] (%)
Y1	Mass loss	18.9	±3.68	61.03	18.5	±3.42	65.3
Y5	L/C loss	1.67	±0.15	80.74	1.61	±0.14	100
Y6	L/H loss	1.79	±0.26	100	1.77	±0.24	73.99
Y7	Y_cellulose[e]	34	±5.47	100	34.14	±5.08	100
Y8	Y_holocellulose[e]	31.93	±4.31	100	31.99	±4.01	99.9
	Global Desirability			83.14			78.44

[a] Estimated values and d_i for Y_6 and are Y_8 provided for information purpose

[b]Estimated values and d_i for Y_5 and are Y_7 provided for information purpose

[c]CI95%: 95% confidence interval for predicted Y_i

[d]d_i: partial desirability of response Y_i

[e]Y_: refers to net carbohydrate conversion yield
